# Rapamycin Regulates Bleomycin-Induced Lung Damage in SP-C-Deficient Mice

**DOI:** 10.1155/2011/653524

**Published:** 2011-03-21

**Authors:** Satish K. Madala, Melissa D. Maxfield, Cynthia R. Davidson, Stephanie M. Schmidt, Daniel Garry, Machiko Ikegami, William D. Hardie, Stephan W. Glasser

**Affiliations:** ^1^Division of Pulmonary Medicine, Cincinnati Children's Hospital Medical Center, 3333 Burnet Avenue, Cincinnati, OH 45229-3039, USA; ^2^Division of Pulmonary Biology, Perinatal Institute, Cincinnati Children's Hospital Medical Center, 3333 Burnet Avenue, Cincinnati, OH 45229-3039, USA

## Abstract

Injury to the distal respiratory epithelium has been implicated as an underlying cause of idiopathic lung diseases. Mutations that result in SP-C deficiencies are linked to a small subset of spontaneous and familial cases of interstitial lung disease (ILD) and interstitial pulmonary fibrosis (IPF). Gene-targeted mice that lack SP-C (*Sftpc* ^−/−^) develop an irregular ILD-like disease with age and are a model of the human SP-C related disease. In the current study, we investigated whether rapamycin could ameliorate bleomycin-induced fibrosis in the lungs of *Sftpc* ^−/−^ mice. *Sftpc* ^+/+^ and −/− mice were exposed to bleomycin with either preventative administration of rapamycin or therapeutic administration beginning eight days after the bleomycin injury. Rapamycin-treatment increased weight loss and decreased survival of bleomycin-treated *Sftpc* ^+/+^ and *Sftpc* ^−/−^ mice. Rapamycin did not reduce the fibrotic disease in the prophylactic or rescue experiments of either genotype of mice. Further, rapamycin treatment augmented airway resistance and reduced lung compliance of bleomycin-treated *Sftpc* ^−/−^ mice. Rapamycin treatment was associated with an increased expression of profibrotic Th2 cytokines and reduced expression of INF-*γ*. These findings indicate that novel therapeutics will be required to treat individuals with SP-C deficient ILD/IPF.

## 1. Introduction

Pulmonary fibrosis is a progressive and often fatal condition characterized pathologically by mesenchymal cell proliferation in the lung, expansion of the extracellular matrix, and extensive remodeling of the lung parenchyma [1]. Lung fibrosis occurs in interstitial lung diseases and idiopathic interstitial pneumonias, as part of several systemic connective tissue diseases and childhood interstitial lung disease syndromes, and in response to many types of lung injury, including radiation and some chemotherapeutic drugs. Idiopathic pulmonary fibrosis (IPF) is perhaps the most intractable form of lung fibrogenesis where the molecular origins are unclear. Recent evidence indicates that the prevalence and mortality of IPF are growing in the U.S. and elsewhere [2]. 

Currently, there are no approved medical antifibrotic therapies for pulmonary fibrosis. Initial therapies focused on aggressive anti-inflammatory treatment; however, this approach has not improved loss of lung function or survival. Pulmonary fibrosis remains a significant public health burden with no proven therapies that prevent or reverse disease progression. As pulmonary fibrosis is likely heterogeneous in molecular etiology, identification of common downstream pathways where signals converge may provide optimal therapeutic targets that will allow treatment of fibrosis regardless of the upstream initiating events. 

Chronic exposures to inhaled particles, infections, and genetic mutations or deficiencies that modify lung function are responsible for persistent inflammation and fibrotic lung diseases. Alveolar type II epithelial cells produce and secrete surfactant lipids and surfactant-associated proteins SP-A, SP-B, SP-C, and SP-D that enhance alveolar compliance and host defense [3]. Recent studies demonstrated a strong association between surfactant protein C gene mutations and familial idiopathic pulmonary fibrosis [4]. SP-C is a small 34 amino acid hydrophobic, alpha-helical protein that is selectively synthesized type II epithelial cells in the lung and secreted into the airspace along with other surfactant lipids. Genetic mutations that can cause misfolding or amyloid fibril formation of SP-C are often associated with interstitial lung disease [5, 6]. In particular, L188Q mutation in SP-C has been shown to associate with human lung disease and found cytotoxic when overexpressed in mouse lung epithelial cells [7]. SP-C-deficiencies are also associated with human lung disease. SP-C deficient mice (*Sftpc* ^−/−^) also developed heterogeneous interstitial lung disease with age [8]. However, molecular mechanisms or pathways that mediate pulmonary inflammation and fibrosis in SP-C deficient mice remained unidentified. 

The mammalian target of rapamycin (mTOR) has been shown to influence tissue fibrosis and is considered a potential control point for pharmacological intervention [9, 10]. In support, inhibition of mTOR with rapamycin has been shown to prevent the initiation and propagation in a mouse model of TGF*α*-driven pulmonary fibrosis [11–14]. The antifibrotic affects of mTOR inhibition have recently been reported in several rat models of chronic kidney disease, including diabetic nephropathy, chronic glomerulosclerosis, and tubulointerstitial fibrosis [15–17]. In rat models of established liver cirrhosis, rapamycin reduced fibrosis and attenuated disease progression [18]. Together, these studies support rapamycin as a potential novel therapy in the treatment of pulmonary fibrosis. Recent recommendations regarding minimal preclinical criteria to be applied before embarking on clinical trials of novel agents in patients with IPF includes demonstration of the antifibrotic effects of the agent in at least two different animal models of lung fibrosis, with drug being delivered during the postinflammatory, fibrogenic phase of lung injury [19]. In this study, we evaluated if rapamycin was effective in reducing bleomycin-induced inflammation and pulmonary fibrosis in the SP-C deficient mice that exhibit an increased response to profibrotic stimuli.

## 2. Methods

### 2.1. Animal Model

*Sf* *tp* *c* ^−/−^ mice were generated by gene targeting techniques. These mice lack SP-C and have been previously described [8]. *Sftpc* ^−/−^ mice were backcrossed onto the 129S6 background to produce a congenic *Sftpc* ^−/−^ 129S6 line of mice that develop lung pathology with age. Mice were maintained in a pathogen-free barrier facility, and sentinel mice were negative for bacterial and viral pathogens. All animals were handled under aseptic conditions. Studies were performed under animal use protocols approved by the institutional animal care and use committee.

### 2.2. Administration of Bleomycin and Rapamycin

Stock bleomycin (Teva Pahramceuticals, North Wales, PA) was prepared by resuspension in sterile PBS (phosphate buffered saline) at 5 U/mL and stored at −80°C. Stock was diluted at time of treatment to 0.5 U/mL and a single 100 ul (0.05 U) delivered by oral aspiration to six-week-old mice that were lightly anesthetized by inhalation of Isoflurane (Abbott Laboratories). Rapamycin (LC Laboratories, Woburn, MA) was prepared as a 30 mg/mL stock in EtOH and diluted to 0.6 mg/mL in 0.25% PEG400/0.25% Tween20. Rapamycin was administered by intraperitoneal (IP) injection of 4 mg/kg of body weight. Rapamycin doses were selected based upon previously effective reduction of injury in experimental lung fibrosis [14]. Control animals were given IP injection of equivalent volumes of the ethanol-detergent dilution vehicle and/or a single oral aspiration of PBS for bleomycin-exposed mice.

### 2.3. Rapamycin Treatment Strategies

Prevention studies were performed to determine if rapamycin administration prior to and throughout the bleomycin-induced fibrosis would prevent or reduce the apparent injury. Daily rapamycin or vehicle IP injections were started two days prior to the single bleomycin exposure and maintained for 14 days following the bleomycin exposure. There were 10 *Sftpc* ^+/+^ and −/− mice in each experimental treatment group. 

Rescue studies were conducted to determine if rapamycin could reduce an evolving fibrotic injury. *Sftpc* ^−/−^ mice received a single 0.05 U bleomycin aliquot by oral aspiration on day 0 and daily rapamycin treatments were begun on day 8 and ended on day 22 after bleomycin. The weights of mice were monitored every two days during both the prevention and rescue experiments. The saline + vehicle and bleomycin + vehicle groups had 8 mice each, and the bleomycin + rapamycin experimental group had 12 mice. Mice were euthanized on day 22 with sodium pentobarbital and lung tissues were collected for RNA, protein, and histological analysis.

### 2.4. Quantification of Bronchoalveolar Lavage-Associated Inflammatory Cells

On day 17, mice were euthanized with sodium pentobarbital (65 mg/kg) and lungs were lavaged sequentially with three 1 mL aliquots of PBS, pooled, and used to determine total cell counts and for cytospin preparations to determine differential cell counts. Cells were isolated from the bronchoalveolar lavage fluid (BALF) by centrifugation at 1250 rpm for 5 minutes, and the cell pellet were resuspended in 1 mL of PBS and cells were counted using hemocytometer. Cell counts were obtained on 5 animals per experimental group. Cell free supernatants were frozen with Roche protease inhibitors, and later, BALF total protein levels were determined using the Pierce bicinchoninic acid (BCA) protein assay kit (Thermo Scientific, Rockford, IL).

### 2.5. Lung Histology

Lungs of *Sftpc* ^+/+^ and −/− mice were collected at day 22 (experimental endpoint) and divided for biochemical and histological analysis. The left lobe was isolated by ligation with surgical suture, removed, and snap frozen for protein and RNA isolation and gene expression analysis. The right lobes were inflation fixed with 10% buffered formalin and removed. Each lobe was bisected, processed, and embedded in paraffin. 5-micron tissue sections were cut and stained with hematoxylin and eosin and with Mason's trichrome stain to visualize morphology and determine the extent and local sites of increased collagen deposition. The lobar airways were scored for the presence or absence of goblet cells as an indication of airway inflammation.

### 2.6. Lung Collagen Determination

Total soluble lung collagen was measured using the Sircol collagen assay (Biocolor Ltd, County Antrim, UK). One half of the left lobe from each animal was homogenized in 5 mL of 0.5 M acetic acid,1 mg pepsin/10 mg tissue and held overnight to solubilize tissue. Commercial Sircol dye reagent was added to tissue lysates (1 mL/100 ul lysate; 30 minutes) and mixtures centrifugedat 12,000 rpm for 12 minutes and the pellets resuspended in 1 mL of 0.5 NaOH and the optical density measured.

### 2.7. Lung Mechanics

Lung mechanics were determined on anesthetized mice using a computerized Flexi Vent Apparatus (SCIREQ Montreal Canada) as previously describe [14]. Briefly, mice were anesthetized with a ketamine-xylazine mixture, tracheostomized, and ventilated with a tidal volume of 8 mL/kg at a rate of 150 breaths/minute and 2 cm of H_2_O positive end expiatory pressure (PEEP) as computerized by the SCIREQ system, thereby permitting analysis of dynamic lung compliance and airway resistance. The calibration procedure removed impedance from the instrument and tracheal tube.

### 2.8. RNA Preparation and Cytokine Gene Expression by Real-Time PCR

Quantitative PCR analysis was used to determine cytokine gene expression. The lung tissues (~20–30 mg) from PBS-treated and bleomycin-treated mice were stored at −80°C. Total RNA was extracted using the SV total RNA isolation system from Promega (Madison, WI) and reverse-transcribed using iScript cDNA synthesis kit (BIO-RAD, Hercules, CA). Real-time polymerase chain reaction (RT-PCR) performed on StepOnePlus Real-Time PCR system (Applied Biosystems, Foster City, CA). Relative quantities of mRNA for several genes were determined using SYBR Green PCR Master Mix (Applied Biosystems, Foster City, CA). In this method, mRNAs for each sample were normalized to hypoxanthine guanine phosphoribosyl transferase (HPRT) mRNA amounts and then expressed as a relative increase or decrease compared with vehicle-treated control mice. Real-time primers used in this study are listed in Table 1.

### 2.9. Statistics

Data was analyzed with Prism (Version 5; GraphPad). Differences between groups were considered statistically significant for *P* values less than  .05, obtained with a one-way ANOVA. Tukey's multiple comparison posttest was used to compare different experimental groups.

## 3. Results

The importance of inflammation in the initiation of experimentally induced fibrosis is unclear and may vary depending upon the specific profibrotic challenge. Early bleomycin response includes a significant inflammatory component prior to fibrosis. The *Sftpc* ^−/−^ mice have an intrinsic pulmonary inflammation, increased response to inflammatory challenge and a delayed or impaired resolution to bleomycin induced fibrosis. Thus, the *Sftpc* ^−/−^ mice are a useful model to assess candidate anti-inflammatory and antifibrotic therapeutics such as rapamycin. Here, the effects of rapamycin were first tested in a preventative scheme prior to and during the developing fibrotic injury followed by studies to discern potential postinjury rescue. 

### 3.1. Early Treatment with Rapamycin Did Not Confer Protection from Bleomycin-Induced Lung Damage in SP-C Deficient Mice

**Figure 1:**
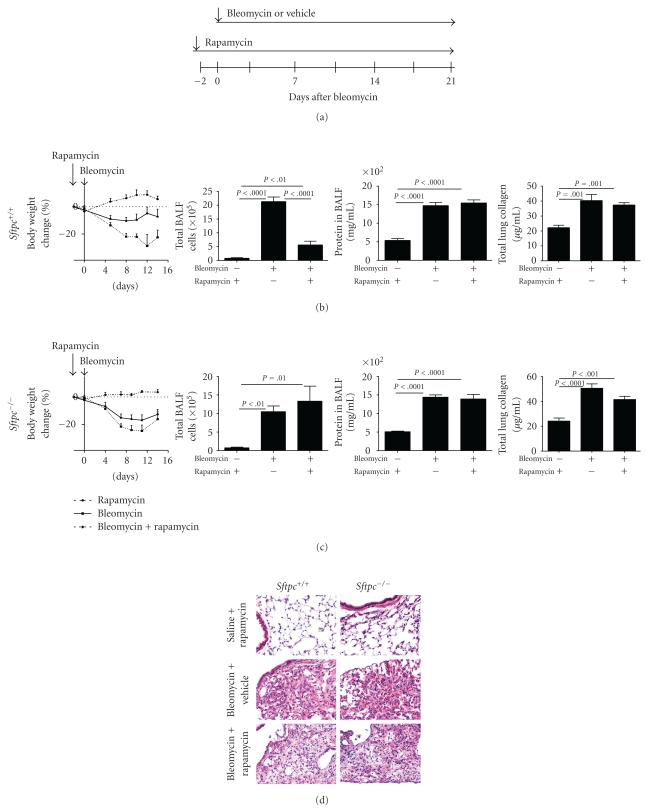
Early rapamycin treatment does not reduce bleomycin-induced acute lung injury in *Sftpc* ^+/+^ or −/− mice. (a) Diagram of experimental protocol for prevention study to test affects of rapamycin on bleomycin-induced lung fibrosis. *Sftpc* ^+/+^ and −/− mice were treated with daily rapamycin or vehicle 2 days prior to administration of intratracheal bleomycin or saline. (b) Changes in body weights, total BALF cells, BALF protein, and total lung collagen are shown fro *Sftpc* ^+/+^ mice. (c) Changes in body weights, total BALF cells, BALF protein, and total lung collagen are shown for *Sftpc* ^−/−^ mice. (d) H&E stained representative lung histology is shown, all panels 40x magnification.

*Sf* *tp* *c* ^+/+^ and *Sftpc* ^−/−^ mice were pretreated two days prior to the bleomycin insult to maximize steady state drug levels and thus the potential to block or reduce the early bleomycin related inflammation (Figure 1(a)). The overall heath and survival was monitored and individual weights within each treatment group were measured. Control animals (rapamycin without bleomycin) remained healthy with a modest weight gain. Both bleomycin only and bleomycin plus rapamycin treated groups had rapid weight loss that began on day four after bleomycin treatment (Figures 1(b) and 1(c)). The bleomycin plus rapamycin-treated mice had a greater weight loss than the bleomycin only challenged group both in *Sftpc* ^+/+^ and −/− mice. This unexpected result was supported by increased mortality beginning day 16 post bleomycin exposure in the bleomycin plus rapamycin treated mice (data not shown). 

Analysis of the BALF from the study groups were used to determine if rapamycin altered the cellular inflammatory response or decreased vascular leak by determining total protein content. Total cell counts in the BALF were increased in the bleomycin treated *Sftpc* ^+/+^ and *Sftpc* ^−/−^ mice relative to control mice. In wild-type mice, bleomycin-induced cellular infiltration is attenuated with rapamycin treatment, but BALF protein levels were unchanged (Figure 1(b)). However, rapamycin treatment has no significant effect on BALF cells or protein levels in *Sftpc* ^−/−^ mice (Figure 1(c)). Rapamycin treatment also has no significant effect on tissue inflammation in both wild-type and *Sftpc* ^−/−^ mice (Figure 1(d)). We measured tissue collagen using sircoll assay to quantify rapamycin driven responses on pulmonary fibrosis. Bleomycin treatment has significantly increased lung collagen in *Sftpc* ^+/+^ (Figure 1(b), right panel) and *Sftpc* ^−/−^ mice (Figure 1(c), right panel). We observed a modest decrease in lung collagen with rapamycin treatment in *Sftpc* ^−/−^ mice; however, the decrease observed was not statistically significant. 

**Figure 2:**
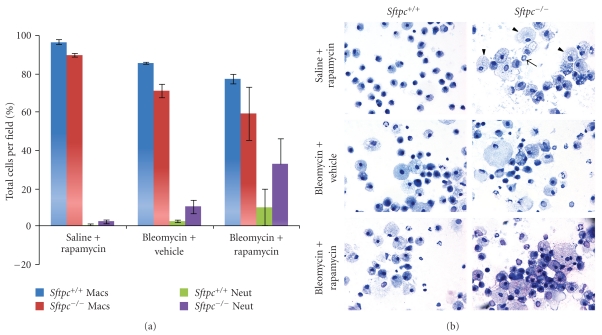
Differential cell counts and morphology of inflammatory cells found in BALF. (a) Macrophages and neutrophils were counted and expressed as a percentage of the total cells counted from cytospin preparations. (b) Representative images of cytospin preparations of BALF cells are shown. Arrowheads identify enlarged foamy macrophages while the arrow indicates a neutrophil for comparison of inflammatory changes among the experimental groups. All images were photographed at 60x magnification.

The cellular component of BALF from control *Sftpc* ^+/+^ mice was predominantly macrophages with normal morphology while the BALF of control *Sftpc* ^−/−^ mice contained enlarged macrophages and a small percentage of neutrophils (Figures 2(a) and 2(b), top panels). The enlarged macrophages and low-level neutrophil presence in BALF of *Sftpc* ^−/−^ mice has been previously reported [8]. Rapamycin treatment alone did not alter the morphology of lavage cells from *Sftpc* ^+/+^ mice (top left panel, Figure 2(b)). Enlarged foamy macrophages and increased numbers of neutrophil were detected in the BALF of bleomycin exposed *Sftpc* ^+/+^ and −/− mice. The rapamycin treatment did not alter/improve the bleomycin-induced foamy macrophage morphology in either genotype (Figure 2(b), lower panels). There was an incremental increase in neutrophils in the BALF of *Sftpc* ^+/+^ mice. The percentage of neutrophils was further increased in the BALF of *Sftpc* ^−/−^ mice (bleomycin exposed and rapamycin treated) and the macrophage morphology contained highly vacuolated foamy macrophages that clustered as aggregates. Airway inflammation was observed by the morphological presence of goblet cells in the airway epithelia of bleomycin and bleomycin-exposed rapamycin treated mice. The extent of goblet cell transformation was more extensive in bleomycin-treated *Sftpc* ^−/−^ mice, and the goblet cell transformation was not reduced by rapamycin treatment (Table 2). Collectively, these results indicated that the presence of rapamycin prior to inflammation did not provide protection to bleomycin-induced lung fibrosis.

### 3.2. Rapamycin Treatment after Bleomycin Challenge Does Not Alter Pulmonary Inflammation and Fibrosis in SP-C-Deficient Mice

A second set of studies were focused on the effects of rapamycin delivered during the postinflammatory, fibrogenic phase of lung injury in *Sftpc* ^−/−^ mice. These experiments were performed in *Sftpc* ^−/−^ mice to evaluate any protective recovery of bleomycin-induced lung damage with rapamycin treatment.

**Figure 3:**
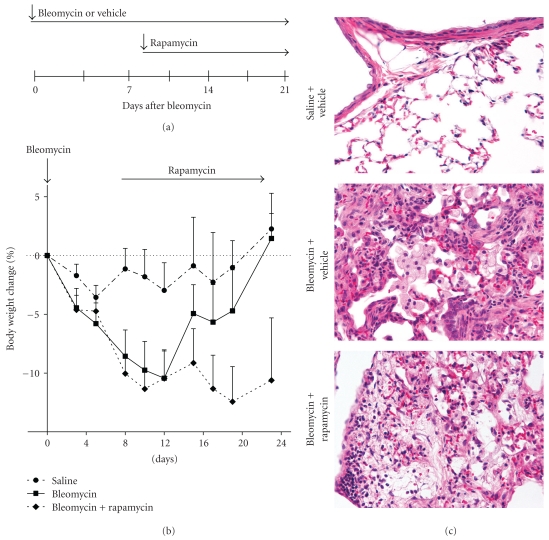
Late rapamycin treatment does not reduce bleomycin-induced weight loss in *Sftpc* ^−/−^ mice. (a) Diagram of experimental protocol for rescue studies to test affects of rapamycin on bleomycin induced lung fibrosis. *Sftpc* ^−/−^ mice were treated with daily rapamycin or vehicle beginning 8 days following administration of intratracheal bleomycin or saline. (b) Body weights in mice receiving bleomycin decreased compared to saline-treated mice. (c) H&E images of mouse lungs 22 days after bleomycin administration. Images in panel (c) are 40x magnification.

*Sf* *tp* *c* ^−/−^ mice were given a single saline or bleomycin challenge and daily rapamycin (4 mg/kg body weight) administered 8 days after bleomycin when inflammation was established (Figure 3(a)). Similar to the prevention study, the control rapamycin only treated mice had a steady weight gain and no mortality. In contrast, the bleomycin and bleomycin plus rapamycin groups had steady decline in body weights beginning 2 days after rapamycin treatment (Figure 3(b)). Weight loss was more severe in the bleomycin plus rapamycin group similar to the trends in the early rapamycin prevention experiments. Survival was again reduced in the bleomycin-exposed mice (data not shown). H&E stained lung sections were analyzed to evaluate tissue inflammation due to bleomycin and rapamycin treatments. Increased tissue inflammation was observed during bleomycin-induced lung injury, and this increase remained unchanged with rapamycin treatment (Figure 3(c)). Cellular infiltrates were mixed including foci of polymorphonuclear cells, lymphocytes, and enlarged foamy macrophages (macrophages visible in Figure 3(c)). These results indicated that the cellular inflammation that occurs early in bleomycin injury was not diminished by delay in the rapamycin treatment. Images are representative of 3–5 mice per treatment group. Collectively, the above results suggest that rapamycin treatment has no protective effects on bleomycin-induced lung inflammation. Moreover, we observe a delay in weight gain upon rapamycin treatments during bleomycin challenge in *Sftpc* ^−/−^ mice.

**Figure 4:**
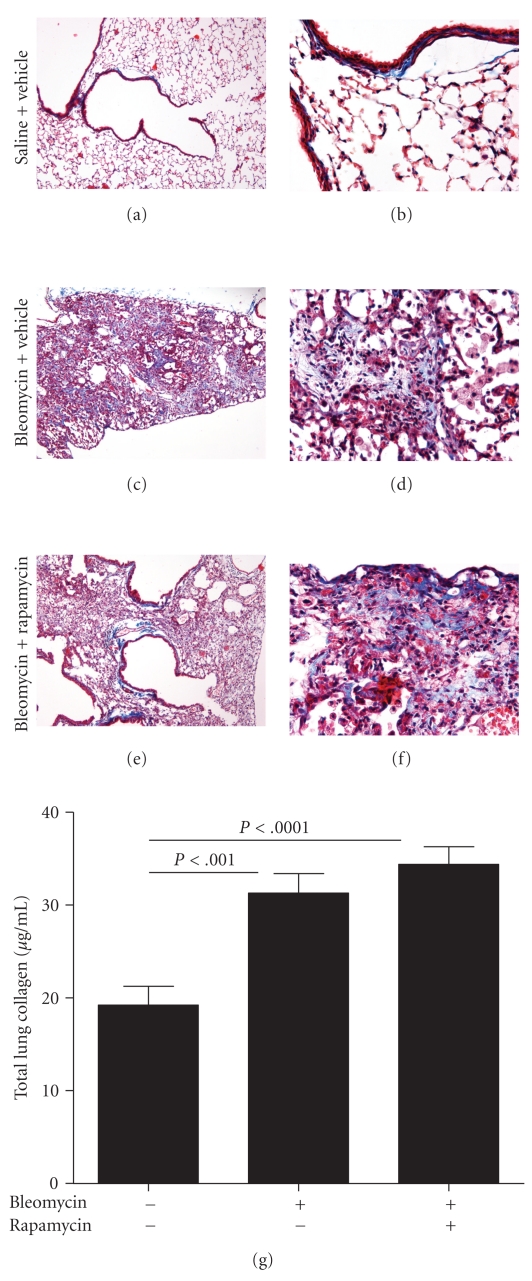
Late rapamycin treatment does not reduce bleomycin-induced pulmonary fibrosis in *Sftpc* ^−/−^ mice. *Sftpc* ^−/−^ mice were treated with daily rapamycin or vehicle beginning 8 days following administration of intratracheal bleomycin or saline. (a)–(f) Mason's trichrome staining demonstrates increased lung matrix deposition in bleomycin-administered mice (c)–(f) compared to saline-treated mice (a, b). (a, c, e) (10x magnification), (b, d, f) (40x magnification). (g) Total lung collagen was increased in mice receiving bleomycin compared to saline-treated mice. There were no differences in total lung collagen in bleomycin mice treated with rapamycin or vehicle.

Pulmonary fibrosis was evaluated by light microscopy of sections stained with Mason's trichrome stain to visualize altered morphology and the potential increase and distribution of tissue collagen within the lungs. Morphology of control mice appeared normal with thin bands of collagen (blue strands) associated in the typical perivascular and peribronchiolar pattern (Figures 4(a) and 4(b)). In contrast, the lungs of bleomycin and the bleomycin plus rapamycin-treated *Sftpc* ^−/−^ mice had extensive disruption of lung architecture and obstruction of alveolar spaces (Figures 4(c) and 4(d)). Extended webs of collagen positive staining areas were present in an irregular pattern throughout the lobe. Fibroblastic-like cellular arrays were seen in the trichrome positive regions at higher magnification (Figures 4(d) and 4(f)). The histological findings were consistent with the biochemical quantification of total lung collagen. Soluble collagen was measured in lung homogenates of the left lungs for each experimental group. Collagen was increased in the lungs of bleomycin plus vehicle mice relative to collagen in the lungs of the saline control group of *Sftpc* ^−/−^ mice (Figure 4(g)). Total lung collagen remained increased in the bleomycin exposed-rapamycin treated *Sftpc* ^−/−^ mice indicating that there was no antifibrotic effects of rapamycin (*n* = 8–12 mice per group).

### 3.3. Rapamycin Treatment Alters Lung Mechanics in Bleomycin-Treated SP-C-Deficient Mice

**Figure 5:**
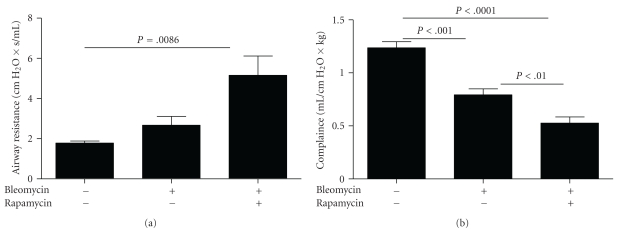
Late rapamycin treatment alters bleomycin-induced changes in the lung mechanics of *Sftpc* ^−/−^ mice. *Sftpc* ^−/−^ mice were treated with daily rapamycin or vehicle beginning 8 days following administration of intratracheal bleomycin or saline. (a) Airway resistance was higher in mice receiving both bleomycin and rapamycin. (b) Compliance was decreased in mice receiving bleomycin compared to saline-treated mice and rapamycin-treated mice demonstrated significantly worse compliance than mice treated with vehicle.

**Figure 6:**
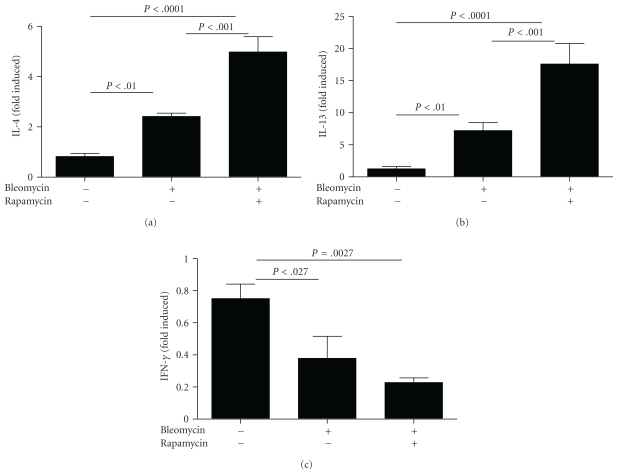
Late rapamycin treatment increases bleomycin-induced increases in IL-4 and IL-13 in *Sftpc* ^−/−^ mice. *Sftpc* ^−/−^ mice were treated with daily rapamycin or vehicle beginning 8 days following administration of intratracheal bleomycin or saline. IL-4, IL-13 and interferon gamma were measured by RT-PCR.

Airway resistance was increased in the lungs of bleomycin-treated *Sftpc* ^−/−^ mice and further increased in the bleomycin plus rapamycin group of *Sftpc* ^−/−^ mice (Figure 5(a)). Airway compliance was reduced in the bleomycin treated mice relative to saline- and vehicle-treated group. Airway compliance was further reduced in the bleomycin plus rapamycin group (Figure 5(b)). Together, these findings indicate that the rapamycin treatment resulted in worsening lung function in bleomycin-treated *Sftpc* ^−/−^ mice (*n* = 8–12 mice per group). However, rapamycin-treated alone has no effect on airway resistance and lung compliance. Th2 cytokines IL-4 and IL-13 have been implicated in altered lung function in various pulmonary disease models and in promoting airway goblet cell hyperplasia [20, 21]. To test whether the sustained injury and poor mechanics were associated with altered Th2 cytokine levels, we measured expression of select Th2 cytokine genes in the lungs from the control and bleomycin +/− rapamycin treated *Sftpc* ^−/−^ mice. IL-4 and IL13 gene expression was increased in the bleomycin treated mice relative to the vehicle on group (Figure 6). We observe significant increases in IL-4 and IL-13 from the lungs of the bleomycin plus rapamycin mice relative to the bleomycin-only-treated group. Furthermore, we also observed attenuation of antifibrotic IFN-*γ* levels in rapamycin and bleomycin treatment compared bleomycin-treated group. These findings are consistent with rapamycin potentiating airway remodeling in bleomycin-treated *Sftpc* ^−/−^ mice (*n* = 4 mice per group).

## 4. Discussion

Using an *Sftpc* ^−/−^ mouse model, the present study demonstrates that administration of rapamycin did not prevent the initiation or the progression of bleomycin-driven pulmonary fibrosis. Rapamycin treatment exacerbated bleomycin-associated alterations in lung mechanics and altered Th2 cytokine gene expression in *Sftpc* ^−/−^ mice.

### 4.1. Altered SP-C Expression Underlies a Genetically Defined Lesion to Investigate a Discrete Form of IPF

Some patients with idiopathic forms of pulmonary fibrosis have a familial pattern of recurrent disease suggesting a genetic component to these cases. The familial forms are of considerable interest regarding the diverse origins attributed to IPF, as they have the potential to reveal a specific gene defect that generates a previously idiopathic disease [5]. The human SP-C gene (*SFTPC*) is one of very few genes whose dysfunction is directly linked to the induction of pulmonary fibrosis. Individuals with a specific *SFTPC* mutation can have extremely variable case histories that range from severe acute infant or early childhood onset of ILD, to progressive ILD or pulmonary fibrosis emerging throughout adulthood [4, 7, 22]. The majority of *SFTPC* mutations cluster in the carboxy-terminal region of the proform of the SP-C protein, altering the proprotein structure and impairing processing. The decreased processing of pro-SP-C also reduces the amount of available mature SP-C in the airspace. There are additional reports of SP-C deficient IPF where there is no mutation in the *SFTPC* sequence and no detectable aberrant pro-SP-C to induce injury [22, 23]. These findings infer that the fibrosis results directly from the absence of SP-C, but the phenotype is complex, where individual severity and ontogeny may be affected by multiple factors.

### 4.2. The SP-C-Deficient Mice Provide a Genetic Model of the Human SP-C-Driven ILD/IPF

To further understand the role of the SP-C protein in human disease, we previously generated SP-C knockout (*Sftpc* ^−/−^) mice. The pulmonary phenotype in *Sftpc* ^−/−^ mice is complex with features similar to human pathology. On the 129S6 genetic background, *Sftpc* ^−/−^ mice exhibited progressive cellular inflammation and interstitial like disease with extensive remodeling, airspace loss and patchy fibrosis in a subset of the older *Sftpc* ^−/−^ mice [8]. 

The 129S6 *Sftpc* ^−/−^ mice also demonstrated an intrinsic increased cellular inflammation including elevated baseline neutrophils and a population of activated alveolar macrophages [8]. Similar findings were seen with the control *Sftpc* ^−/−^ mice in this study that had slightly elevated neutrophil counts and foamy macrophages in the BALF prior to any bleomycin challenge. When the 129S6 *Sftpc* ^−/−^ mice were challenged by bacterial or viral infection, mice had increased injury that included robust unremitting inflammation relative to *Sftpc* ^+/+^ mice [24]. Viral and bacterial infections have also been associated with acute respiratory illness in individuals with *SFTPC* mutations implicating SP-C in innate lung defense [4, 7, 22]. The mechanism whereby SP-C deficiency predisposes the lung to inflammation is unknown and remains under investigation. SP-C has been shown to bind to LPS and block TLR signaling in vitro, suggesting SP-C regulates proinflammatory responses to acute injury.

### 4.3. The *Sftpc* ^−/−^ Mice Are More Sensitive to Bleomycin-Induced Inflammation

We previously demonstrated that *Sftpc* ^−/−^ mice on a Swiss/Black (S/B) mixed genetic background do not display any intrinsic lung pathology yet were more susceptible to lung injury following challenge with bleomycin [25]. The S/B *Sftpc* ^−/−^ mice had an increased and sustained neutrophil influx relative to S/B *Sftpc* ^+/+^ mice administered bleomycin. S/B *Sftpc* ^−/−^ mice also demonstrated increased collagen deposition with delayed resolution compared to *Sftpc* ^+/+^ mice. In the current, study inflammation was sustained in the lungs of 129S6 *Sftpc* ^−/−^ mice compared to 129S6 *Sftpc* ^+/+^ mice with bleomycin treatment until the termination at day 22. On the 129S6 background, the *Sftpc* ^−/−^ mice eventually develop ILD-like lung pathology [8]. Airway epithelial inflammation was increased by bleomycin treatment supporting the concept that SP-C confers protection to the airway epithelium (Table 2). Airway inflammation quantified by the presence of goblet cells was increased in 129S6 *Sftpc* ^−/−^ mice relative to *Sftpc* ^+/+^ mice, and the goblet cell hyperplasia was not reduced by rapamycin treatment. While SP-C is expressed in the alveolar epithelium, SP-C is detected in tracheal aspirates of infants and thus a component of airway lining fluid. In addition, the cellular inflammation in the BALF of *Sftpc* ^−/−^ mice was not altered by rapamycin treatment. Taken together, these findings are consistent with SP-C functioning to limit alveolar and airway epithelial inflammation.

### 4.4. Rapamycin Has No Effect on Bleomycin-Induced Tissue Fibrosis

Rapamycin binds to an intracellular cytoplasmic receptor, the FK506-binding protein-12 [11, 15]. This complex interacts and inhibits mTOR function leading to cell-cycle arrest in the G1 phase. Rapamycin derivates sirolimus and everolimus are utilized clinically primarily as an immunosuppressive agent following organ transplantation, but has also been investigated as an antineoplastic agent and as adjunctive therapy in systemic diseases [15–18]. In pulmonary fibrosis models, there is limited data on activation of mTOR and the effectiveness of rapamycin treatment. Rapamycin prevented fibrosis in transgenic mice overexpressing transforming growth factor-alpha (TGF*α*) as well as prevented progression of fibrosis when rapamycin was administered after extensive fibrosis had already developed. Similar to the TGF*α* model, the rapamycin analog SDZ RAD prevented bleomycin-induced pulmonary fibrosis in rats although it was unclear whether changes in lung inflammation may have contributed to these improvements [26]. Notably, in this model, histological evaluation failed to show a significant difference in apparent fibrosis between animals receiving bleomycin alone and those receiving bleomycin and SDZ RAD. In another study by Mehrad et al. using C57BL/6, mice also reported antifibrotic effects of rapamycin [27]. However, both of these studies used a different species or strain of mice and also low doses of rapamycin. Moreover, the 129 strain of mice are known to be sensitive to bleomycin induced injury. In gene microarray experiments following bleomycin treatment, the 129 strain of mice were shown to have a distinct expression profile that overlapped with the expression profile of bleomycin challenged C57/BL6 mice [28]. It is also possible that partial inhibition of mTOR signaling with a low dose of rapamycin may be protective against bleomycin-induced fibrosis. Therefore, future studies will be needed to evaluate internal and external factors that may contribute to mTOR pathway activation and antifibrotic doses of rapamycin. These studies will be useful to address differences in fibrosis associated with a specific genetic background of mice and doses of rapamycin in bleomycin-driven fibrosis. To date, there are no published clinical trials testing the efficacy of mTOR inhibition in patients with pulmonary fibrosis; however, there is one case report describing partial remission of IPF in a patient treated with rapamycin [29]. 

As rapamycin has both immunomodulatory and antifibrotic properties, we hypothesized rapamycin would be an effective intervention to amend the fibrosis and exacerbated inflammation in the *Sftpc* ^−/−^ bleomycin model. In the current study, rapamycin was ineffective in reducing inflammation and fibrosis when administered either as a preventative pretreatment regimen or delayed until 8 days following the onset of bleomycin injury in *Sftpc* ^−/−^ mice. However, rapamycin was effective in reducing inflammatory cells in BALF of 129S6 *Sftpc* ^+/+^ mice. The lungs of *Sftpc* ^−/−^ mice with or without daily rapamycin treatments retained collagen rich tissue as well as irregular mixed inflammatory cell infiltrates. Rapamycin-treated *Sftpc* ^−/−^ mice demonstrated persistent weight loss, and both airway resistance and compliance were further compromised. The goblet cell hyperplasia in bleomycin exposed *Sftpc* ^−/−^ mice both with and without rapamycin may in part account for the impaired lung mechanics.

### 4.5. Rapamycin Alters Cytokine Gene Expression in the Lungs of Bleomycin-Exposed *Sftpc* ^−/−^ Mice

At day 22 after bleomycin, both diffuse and discrete regional concentrations of polymorphonuclear leukocytes, lymphocytes, and enlarged foamy macrophages were observed. The ongoing mixed cellular alveolitis in the treated and untreated *Sftpc* ^−/−^ mice was not reduced by rapamycin and may in part be due to sustained cytokine activation. Bleomycin stimulated expression of IL-4 and IL13 relative to the control mice. Expression was further increased in the rapamycin plus bleomycin group of *Sftpc* ^−/−^ mice. Elevated Th2 cytokines might be responsible for observed increases in airway remodeling due to rapamycin treatment during bleomycin-induced lung damage. 

Differential effects of rapamycin on cytokines levels are also observed in other models of tissue damage and repair. In particular, rapamycin has been shown to polarize the Th2 type cellular response in vitro that was dependent upon IL-4 [30]. IL-13 contributes to induction of bleomycin mediated fibrosis in mice [31]. A mechanism by which rapamycin exerts the hypothesized Th2 bias in SP-C deficient mice is unknown but is consistent with the cellular influx and airway inflammation reported herein. In addition bacterial and viral challenges of *Sftpc* ^−/−^ mice have resulted in an increased goblet cell hyperplasia that is a Th2-dependent process [24, 32]. Future studies will be needed to determine the phenotype of the inflammatory cells recruited into the lungs of injured *Sftpc* ^−/−^ mice.

### 4.6. Rapamycin Associated Pulmonary Toxicity in Humans

Our findings of deteriorated lung function in rapamycin-treated *Sftpc* ^−/−^ mice may provide insight into the known and potentially devastating toxicity observed in patients. Pulmonary toxicity from mTOR inhibitors such as sirolimus and everolimus has been reported in up to 15% of patients with a wide spectrum of disease severity, ranging from subclinical to fulminant respiratory failure [33, 34]. Several distinct types of pulmonary damage have been recognized, including lymphocytic interstitial pneumonitis, lymphocytic alveolitis, bronchiolitis obliterans with organizing pneumonia, pulmonary alveolar proteinosis, alveolar hemorrhage, focal pulmonary fibrosis, or a combination of these entities [33–36]. Treatment includes dose reduction or withdrawal of medication and corticosteroids are administered in severe cases [34, 36]. Clinical improvement with resolution of pulmonary findings is usually rapid although protracted resolution is reported in severe cases of pneumonitis [36].

The pathogenic mechanism of pulmonary toxicity associated with mTOR inhibition is not well understood. Both a direct, dose-dependent toxicity and an autoimmune response or delayed hypersensitivity reaction triggered by exposure to mTOR inhibitors with or without a cryptic pulmonary antigen have been postulated as possible underlying pathogenetic mechanisms [34]. mTOR inhibition has also been hypothesized to hamper cellular repair in alveolar cells by the inhibition of growth factor-driven signal transduction response, thereby exacerbating epithelial injury [35, 37]. 

Potential clinical relevance is that patients with acute lung injury from a variety of causes such as infection or ventilation often produce less or transiently shut off SP-C production [38]. Our data demonstrates that in the absence of SP-C, rapamycin treatment following acute lung injury may exacerbate the immune response by shifting to a Th2 response. 

In summary, to date, each defined genetic modifier of acute lung injury and fibrosis occurs through functionally unrelated cellular events. For instance, mutations in the telomerase genes alter either the RNA required to primer telomere repeat replication or the enzyme that extends the repeats. Mutations in the pulmonary collectin, SP-A has been associated with familial fibrosis and susceptibility to infection. Hermansky Pudlak gene mutations alter vesicle traffic proteins and are linked to sporadic inflammation and lung fibrosis with age. Thus, it may be that each mutation-cellular deficiency that eventually elicits inflammation and fibrosis will require individualized inhibitor design. And only a very small subset of idiopathic lung disease the focus on *SFTPC*-related IPF will aid in defining cellular and molecular alterations that provide insights to other undefined forms of IPF and their inhibitors.

## Figures and Tables

**Table 1:** Real-time primers used in the study. The fold change was obtained by normalizing the gene expression number to those of HPRT, then comparing the samples to the PBS-treated or control mice.

Gene	Forward	Reverse
HPRT	GCCCTTGACTATAATGAGTACTTCAGG	TTCAACTTGCGCTCATCTTAGG
IL-13	CCTCTGACCCTTAAGGAGCTTAT	CGTTGCACAGGGGAGTCTT
IL-4	ACGAGGTCACAGGAGAAGGGA	AGCCCTACAGACGAGCTCACTC
IFN-*γ*	AGAGCCAGATTATCTCTTTCTACCTCAG	CCTTTTTCGCCTTGCTGTTG

**Table 2:** Goblet cell positive large airways (secondary bronchi).

Treatment	*Sf* *tp* *c* ^+/+^	*Sf* *tp* *c* ^−/−^
PBS + rapamycin	0/23	0/16
Bleomycin + vehicle	3/25 (12)	6/19 (32)
Bleomycin + rapamycin	0/10	4/13 (31)

( ) % positive airways.
